# High Levels of Multiple Infections, Recombination and Horizontal Transmission of *Wolbachia* in the *Andricus mukaigawae* (Hymenoptera; Cynipidae) Communities

**DOI:** 10.1371/journal.pone.0078970

**Published:** 2013-11-08

**Authors:** Xiao-Hui Yang, Dao-Hong Zhu, Zhiwei Liu, Ling Zhao, Cheng-Yuan Su

**Affiliations:** 1 Laboratory of Insect Behavior and Evolutionary Ecology, Central South University of Forestry and Technology, Changsha, China; 2 Department of Biological Sciences, Eastern Illinois University, Charleston, Illinois, United States of America; University of Sussex, United Kingdom

## Abstract

*Wolbachia* are maternally inherited endosymbiotic bacteria of arthropods and nematodes. In arthropods, they manipulate the reproduction of their hosts to facilitate their own spread in host populations, causing cytoplasmic incompatibility, parthenogenesis induction, feminization of genetic males and male-killing. In this study, we investigated *Wolbachia* infection and studied *wsp* (*Wolbachia* surface protein) sequences in three wasp species associated with the unisexual galls of *A. mukaigawae* with the aim of determining the transmission mode and the reason for multiple infections of *Wolbachia*. Frequency of *Wolbachia* infected populations for *A. mukaigawae*, *Synergus japonicus* (inquiline), and *Torymus* sp. (parasitoid) was 75%, 100%, and 100%, respectively. Multiple *Wolbachia* infections were detected in *A. mukaigawae* and *S. japonicus*, with 5 and 8 *Wolbachia* strains, respectively. The two host species shared 5 *Wolbachia* strains and were infected by identical strains in several locations, indicating horizontal transmission of *Wolbachia.* The transmission potentially takes place through gall tissues, which the larvae of both wasps feed on. Furthermore, three recombination events of *Wolbachia* were observed: the strains W_8_, W_2_ and W_6_ apparently have derived from W_3_ and W_5a_, W_6_ and W_7_, W_4_ and W_9_, respectively. W_8_ and W_2_ and their respective parental strains were detected in *S. japonicus*. W_6_ was detected with only one parent (W_4_) in *S. japonicus*; W_9_ was detected in *Torymus* sp., suggesting horizontal transmission between hosts and parasitoids. In conclusion, our research supports earlier studies that horizontal transmission of *Wolbachia*, a symbiont of the Rickettsiales order, may be plant-mediated or take place between hosts and parasitoids. Our research provides novel molecular evidence for multiple recombination events of *Wolbachia* in gall wasp communities. We suggest that genomic recombination and potential plant-mediated horizontal transmission may be attributable to the high levels of multiple *Wolbachia* infections observed in *A. mukaigawae* and *S. japonicus.*

## Introduction

*Wolbachia* are maternally inherited endosymbiotic bacteria of the family of Anaplasmataceae and infect filarial nematodes and arthropods, including insects, mites, spiders and isopods [1], [2]. In arthropods, these intracellular bacteria can manipulate host reproduction to enhance their own spread throughout host populations, causing cytoplasmic incompatibility, parthenogenesis induction, feminization of genetic males and male-killing [3]. Recent survey of *Wolbachia* incidence estimated that 66% of insect species are infected with *Wolbachia* [4]. Furthermore, a number of studies have shown that multiple strains of *Wolbachia* can infect a host species in various insect groups, *e.g.,* parasitic wasps [5], fruit flies [6], ants [7], [8], gall wasps [9], beetles [10], [11], and butterflies [12]. It is difficult to explain multiple *Wolbachia* infection by vertical transmission alone, and obviously some species can acquire new infection through horizontal transmission and recombination [7].

Vertical transmission from parents to offspring through the egg cytoplasm has previously been considered to be the main transmission mode of *Wolbachia* [2]. However, phylogenetic incongruence between hosts and their *Wolbachia* suggests *Wolbachia* horizontal transmission among arthropods [5], [13]. Laboratory studies demonstrated that *Wolbachia* were transmitted artificially from infected to uninfected individuals by microinjection [14]–[17]. One particular also reported frequent observation of *Wolbachia* being naturally transmitted horizontally from infected to uninfected wasp larvae sharing a common butterfly egg [18].

The four known mechanisms of horizontal transmission of *Wolbachia* include host-parasitoid association, blood contact, feeding relationship, and common usage of same plant tissues. Transmission through host-parasitoid relationship is probably the main form of horizontal transmission. Several studies have found the host and the parasitoid to be infected by the same *Wolbachia* strain in *Drosophila*-parasitoid communities [13], [19], [20]. Brief contact of the blood between infected and wounded uninfected woodlice had obviously caused horizontal transmission [21]. Infection through feeding relationship was suggested by the observation that closely related *Wolbachia* strains in hoppers and mirid bugs feeding on the same hopper eggs [22]. Plant tissue mediated horizontal transmission of *Wolbachia* was supported by several studies. One study reported that individuals of two leafhopper species feeding on the same mulberry leaf substrate were infected with the same *Wolbachia* strain [23]. Another found that four taxonomically diverse insect species feeding on the same pumpkin leaf substrate harbored the same *Wolbachia* strain [24]. In addition, it has been demonstrated that insect symbiotic *Rickettsia* could be transferred from whiteflies *Bemisia tabaci* to the phloem of the leaves of host plants, and be subsequently acquired by other *B. tabaci* individuals [25].

Recombination in *Wolbachia* was considered to be rare or nonexistent [2], but recent studies have indicated that recombination in *Wolbachia* is by no means as rare as previously thought. The first case of recombination was reported between two *Wolbachia* strains in a fly host and its parasitoid wasps [26]. Additional recombination events of *Wolbachia* were later observed in *Armadillidium vulgare* [27], *Byturus tomentosus* [11], *Formica exsecta* [7], and *Rhagoletis cerasi* [28]. Moreover, recombination may occur between *Wolbachia* strains belonging to different supergroups [11], [29], [30].

Cynipidae (Hymenoptera, Cynipoidea) is a phytophagous insect family consisting of six tribes, *i.e.,* Aylacini, Eschatocerin, Diplolepidini, Pediaspidili, Cynipini, and Synergini [31]. Species of Aylacini, Eschatocerini, Diplolepidini, Pediaspidili, and Cynipini are all gall makers and induce structurally complex and morphologically diverse galls on the host plants. In contrast, species of Synergini are mostly inquiline and are unable to induce gall, but live as obligate guest inhabitant of the gall induced by the gall-inducing host, deriving all nutrients from the gall tissue initiated by the host [32]. *Wolbachia* are known to infect quite a number of species in the gall inducing tribes of Aylacini [33], Diplolepidini [34], [35], and Cynipini [36]–[38], as well as in Synergini [9].

Cynipid galls are modified plant structures consisting of live tissues and are fed on by both the gall inducer and the inquiline wasps inhabiting the same galls, and thus can potentially serve as media for horizontal transmission of *Wolbachia* between the two. Frequently a large number of parasitic wasps, often belonging to multiple species, were reared from cynipid galls [39], [40]. These parasitic wasps may parasitize either one or both kinds of the gall inhabitants, potentially allowing for horizontal transmission of *Wolbachia* between the parasitoids and their hosts. A combination of the two horizontal transmission mechanisms, host-parasitoid association and common usage of the same plant tissues, may result in the infection of same *Wolbachia* strains across all three trophic levels and multiple infections of the same *Wolbachia* strains in some individuals of any of the three species, allowing for genomic recombination between strains. Therefore, cynipid galls and their associated insect communities form ideal systems for studying horizontal transmission of *Wolbachia* between involved species [9] and genomic recombination between *Wolbachia* strains.

*Andricus mukaigawae* of the tribe Cynipini is widely known from Japan, Korea, Russia and India [41], [42]. Like many species of Cynipini, *A. mukaigawae* exhibits heterogony, with alternating bisexual and unisexual generations, which induce galls on host leaves and buds, respectively [41]. Very high *Wolbachia* infection rates were reported for the unisexual generation in two populations of *A. mukaigawae* in Hinoharu and Nose, Japan [36]. The unisexual galls of *A. mukaigawae* are also inhabited and fed on by the inquiline species *Synergus japonicus* [43]. In addition, a parasitic species, *Torymus* sp. (Torymidae, Chacidoidea, Hymenoptera), was reared from the unisexual galls, although it is not clear whether it attacks the gall inducer or the inquiline, or both. Nonetheless, *S. japonicus* and *Torymus* sp. were not previously known to be infected by *Wolbachia*, and hence horizontal transmission was unheard of in the system.

In this study, we investigate the possible horizontal transmission of *Wolbachia* between *A. mukaigawae* (gall inducer), *S. japonicus* (inquiline) and *Torymus* sp. (parasitoid) associated with the unisexual galls of *A. mukaigawae.* We employed a PCR-based method with *Wolbachia* specific gene markers [44] for detection of *Wolbachia* infection in the adults of all three species reared from galls collected from eight locations in southern China. We subsequently sequenced the detected *Wolbachia* isolates and compared the sequences to study the possible relationships among them. We found *Wolbachia* infection in all three species, and identified nine distinct *Wolbachia* strains in the various populations of the sampled wasp communities. We then discussed the implications of our findings for the high levels of multiple *Wolbachia* infection observed in the *A. mukaigawae* communities.

## Materials and Methods

### Ethics Statement

The sampling of living material involved in our experiments included three species of wasps, i.e., *A. mukaigawae*, *S. japonicus*, and *Torymus* sp., associated with galls on the oak species *Quercus fabri* Hance. All sampling locations are not privately owned or protected. Neither the oak nor the wasp species are endangered or otherwise protected, and therefore no specific permits were required for these locations/activities.

### Collection and Rearing of Galls

**Figure 1:**
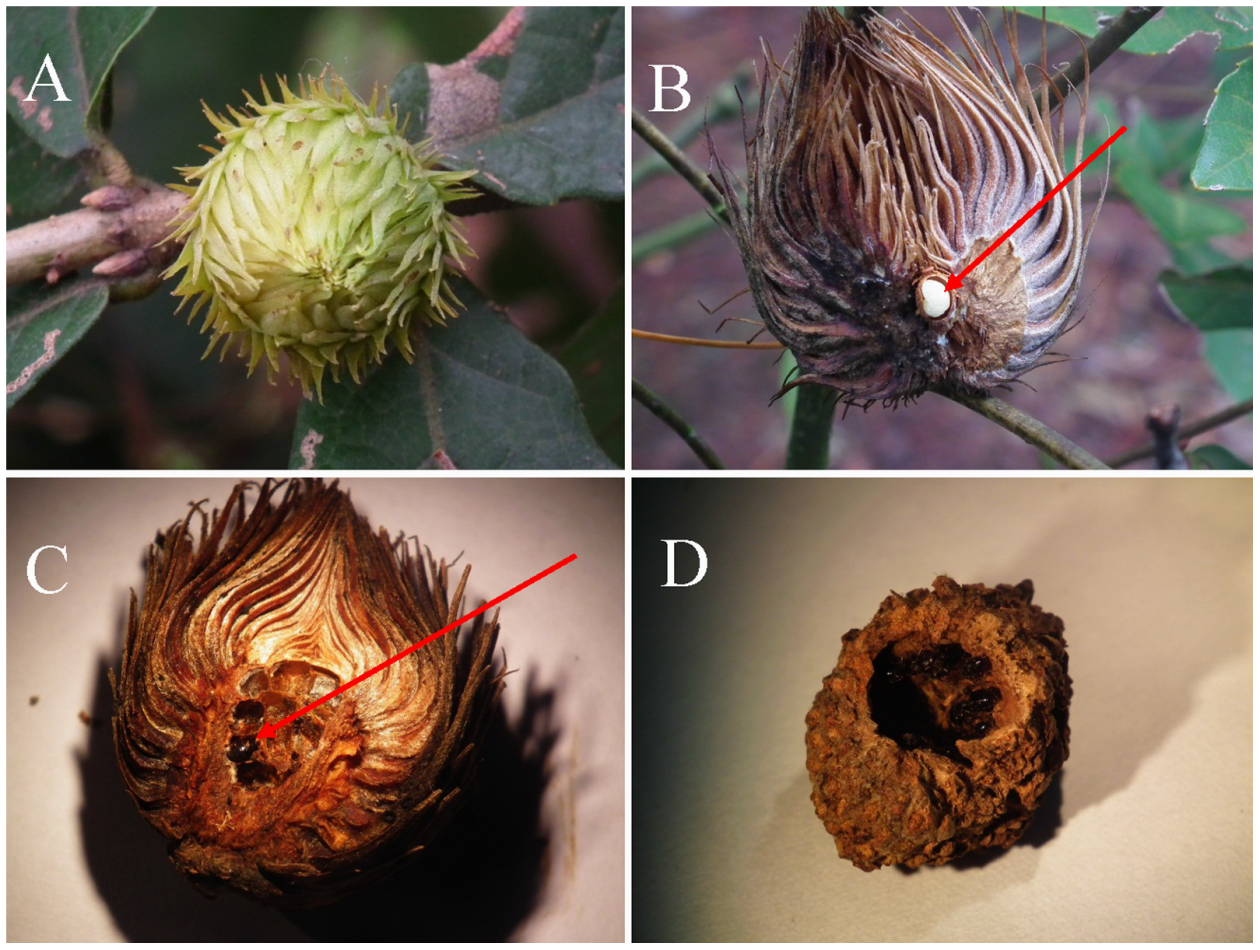
The galls of *A. mukaigawae* on *Quercus fabri* Hance. A: Young gall. B: Monolocular unisexual gall of *A. mukaigawae*; arrow points to the larva of *A. mukaigawae.* C: Multilocular gall of *S. japonicus*; arrow points to the adults of *S. japonicus*. D: Gall destroyed by various insects.

Unisexual galls of *A. mukaigawae* on *Quercus fabri* Hance were collected from eight locations in southern China through September–December in 2010 and 2011 (Table 1 and Figure 1). The galls were reared in a climate chamber set at room temperature. Adults of *A. mukaigawae* emerged in December 2010 through February 2011 and in December 2011 through February 2012. *S. japonicus* and *Torymus* sp. emerged in mid-March through early May, 2012. All adults were killed live within 2 days after emergence and preserved in 100% ethanol at −40°C.

**Table 1:** Sample location, gall types, sex ratio and infection frequency of *Wolbachia* in three wasp species associated with the unisexual galls of *A. mukaigawae.*

Location (code)	Latitude/Longitude	No. of gall(Type I/II/III †)	Species	No. offemale/male	Infectionfrequency of *Wolbachia* (%)	No. ofindividuals tested
Anqing, Anhui (AQ)	30. 42°N/116. 27°E	148(70/29/49)	*A. mukaigawae* ‡	52/0	30	30
			*S. japonicus*	118/32	60	30
			*Torymus* sp.	18/6	100	10
Changde, Hunan (CD)	29. 58°N/111.38°E	7(5/0/2)	*A. mukaigawae*	5/0	0	5
			*S. japonicus*	–‡	–	–
Yueyang, Hunan (YY)	29. 15°N/113. 12°E	77(30/24/23)	*A. mukaigawae*	27/0	40	15
			*S. japonicus*	49/25	50	30
Changsha, Hunan (CS)	28. 06°N/112. 52°E	29(18/9/2)	*A. mukaigawae*	18/0	40	15
			*S. japonicus*	74/18	60	25
Loudi, Hunan (LD)	27. 45°N/112. 20°E	93(30/43/20)	*A. mukaigawae*	29/0	33	15
			*S. japonicus*	70/29	60	20
Shaoyang, Hunan (SY)	26. 37°N/110. 32°E	118(68/29/21)	*A. mukaigawae*	62/0	40	25
			*S. japonicus*	73/40	70	20
			*Torymus* sp.	26/8	100	10
Jian, Jiangxi (JA)	26. 34°N/114. 10°E	50(17/19/14)	*A. mukaigawae*	12/0	0	10
			*S. japonicus*	60/20	60	25
Shaoguang, Guangdong(SG)	25. 13°N/113. 35°E	48(35/8/5)	*A. mukaigawae*	29/0	40	20
			*S. japonicus*	33/7	60	25

† Gall types, I: *A. mukaigawae* galls; II: *S. japonicus* galls; III: other galls.

‡ indicates no wasp was reared.

‡ *A. mukaigawae* is the gall-inducer and the host, *S. japonicas* is an inquiline, or gall parasite, and Torymus sp. is a parasitoid species, whose exact host is not clear, may be either of *A. mukaigawae* or *S. japonicas,* or both.

### DNA Extraction and *Wolbachia* Screening

5–30 female adults of each of the three wasp species were picked randomly for each local gall associated community for detection of *Wolbachia* infection (Table 1). The insects were washed in sterile water to avoid cross-contamination before DNA extraction. Total DNA was extracted from each individual using SDS/proteinase K digestion and phenol-chloroform extraction method as previously described [37].

We screened for *Wolbachia* by polymerase chain reaction (PCR) with the *Wolbachia*-specific primers *wsp*-81F and *wsp*-691R that amplify a 575–625 bp fragment of *wsp* gene encoding *Wolbachia* surface protein [44]. The amplification was conducted using a MJ thermal cycler in a 50 µl reaction volume: 5 µl 10× PCR buffer, 1 µl Taq polymerase (2 U/µl), 2 µl dNTPs (2.5 mM each), 2 µl forward and reverse primer (10 µM), 2 µl DNA template and 36 µl sterile water. The *wsp*-PCR cycling conditions were: 5 min at 95°C, followed by 35 cycles of 30 s at 95°C, 45 s at 52°C, 1 min at 72°C, and a final elongation step of 15 min at 72°C. All PCR screenings were performed with positive and negative controls to check for contamination. Positive samples were prepared from known *Wolbachia*-infected strains of *Dryocosmus kuriphilus* [37]. Negative samples were *Wolbachia*-free strains of *D. kuriphilus* and ddH_2_O. 5 µl of PCR products were run on 1% agarose gel and electrophoresis was performed in 0.5× TBE buffer. Gels were stained with Ethidium Bromide and visualized under UV transilluminator.

### Sequencing and Phylogenetic Analyses

Three individuals were picked from each *Wolbachia*-positive population to sequence the *wsp* fragment directly from purified PCR products using PCR primers. The appearance of multiple peaks in a sample through initial sequencing was taken as indication of multiple *Wolbachia* infection, and the *wsp* fragments were then sequenced for 10 different clones of the same sample to confirm multiple *Wolbachia* infection. To do this, PCR products of the *wsp* gene were purified using the V gene gel extraction kit and ligated directly into the pMD18-T cloning vector (BGI tech., Beijing, China) following the manufacturer’s protocols. For each sample, 3–5 independent positive colonies were isolated and cultured in Lysogeny broth (LB) medium fortified with ampicillin. Plasmids were extracted and partially sequenced in both directions using an ABI 377 DNA sequencer with M13F/R and BigDye Terminator sequencing ready kit (BGI tech, Beijing, China).

The final sequence dataset consisted of 180, 210, and 60 *wsp* sequences from *A. mukaigawae*, *S. japonicas*, and *Torymus* sp., respectively. All *wsp* sequences were aligned together using the CLUSTAL X with default settings [45]. The third hypervariable region (518–581 bp) of the sequences was removed from the analysis because it could not be confidently aligned [44], [46]. Modeltest version 2.1 was used to find the most appropriate model of molecular evolution via the Akaike Information Criterion [47]. The GTR+G model was selected as the best fit model for the dataset. The dataset was then analyzed using maximum likelihood (ML) method implemented in PAUP 4.0 b [48]. A neighbor-joining (NJ) tree was also generated in MEGA 4.0 [49] using Kimura 2-parameter distance model and gamma shape parameters chosen by Modeltest. Both ML and NJ trees were subjected to 1000 bootstrap replicates to assess branch support.

### Typing and Genetic Distances of *Wolbachia* Strains

Genetic distances between all sequence pairs were calculated using Kimura 2-parameter distance model with Complete Deletion option in MEGA 4.0. *Wolbachia* strains with genetic distances smaller than 1.5% were defined as identical strains according to previously described method [24], [44]. The 9 distinct *wsp* sequences identified were designated as W_1_–W_9_, and were deposited in GenBank under accession number KC130968–KC130977.

### Recombination Analysis of *Wolbachia* Strains

Six recombination detection methods implemented in the RDP3 program [50] were for identification of recombinant sequences and breakpoints: RDP, GENECONV, BootScan, Maxchi, Chimaera, and 3Seq. The default settings were used for all methods, and the highest acceptable P value cut-off was set to 0.05. The third hyper variable region of the *wsp* sequences was included in all analyses.

## Results

### *Wolbachia* Infection in *A. mukaigawae* Communities

A total of 570 unisexual galls of *A. mukaigawae* were collected from eight locations during September–December 2010 and 2011. The galls were classified into three types: (I) normal *A. mukaigawae* galls with single larval chamber; (II) *S. japonicus* modified galls with multiple larval chambers; (III) undeveloped small galls and galls destroyed by various insects (Table 1 and Figure 1).

A total of 234 *A. mukaigawae* females were acquired from eight populations. Six out of the eight sampled populations (75%) of *A. mukaigawae* were infected with *Wolbachia*, with infection frequencies varying between 30% and 40%; the two uninfected populations were from Changde and Jian. A total of 477 females and 171 males of *S. japonicus* were reared from seven of the eight locations. All seven populations of *S. japonicus* (100%) were infected with *Wolbachia*, with infection frequencies varying between 50% and 70%. 44 females and 14 males of *Torymus* sp. were reared from galls collected in Anqing and Shaoyang, with 100% *Wolbachia* infection frequency.

### Typing and Multiple Infections of *Wolbachia* in *A. mukaigawae* Communities

**Figure 2:**
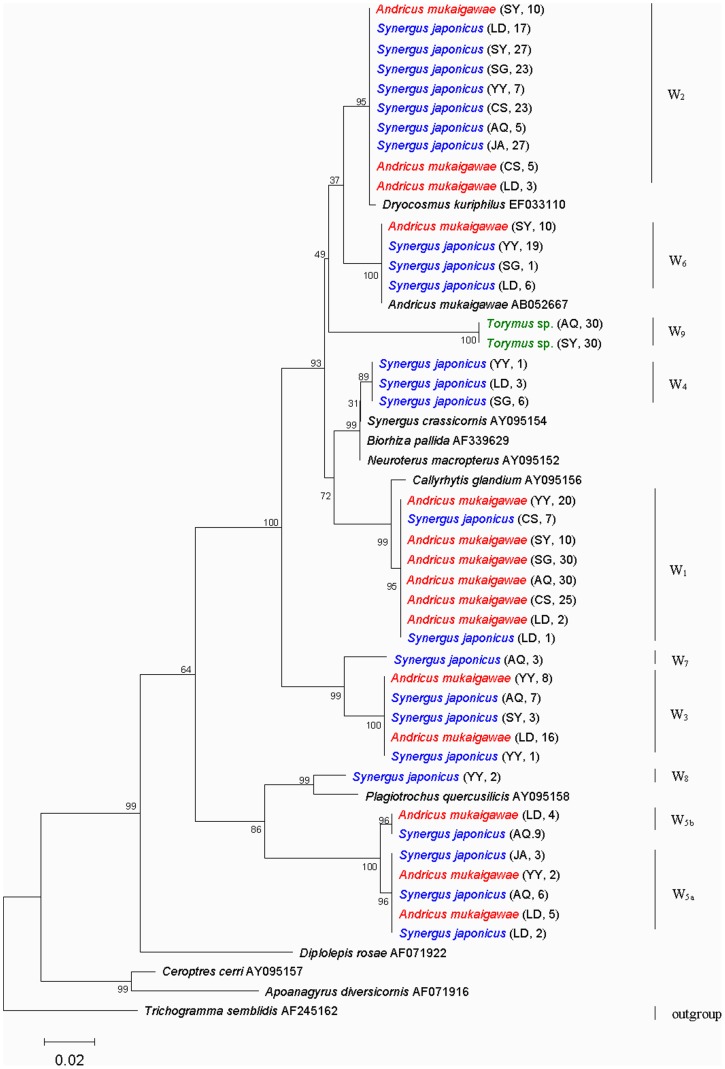
Neighbor-joining tree for *Wolbachia* strains of *A. mukaigawae, S. japonicus* and *Torymus* sp. based on *wsp* sequence. *A. mukaigawae, S. japonicus* and *Torymus* sp. are shown in red, blue and green, respectively. W_1_–W_9_ represent *Wolbachia* strains in *A. mukaigawae* communities. The abbreviations AQ, YY, CS, LD, SY, JA and SG in parentheses indicate the sampled populations shown in Table 1. The number following AQ, YY, CS, LD, SY, JA and SG indicate the amount of *Wolbachia* strains per population. Numbers above branches are bootstrap values computed from 1000 replications. *Wolbachia* from *Trichogramma semblidis* was used as outgroup.

We acquired a total of 450 *wsp* sequences, including 180 from *A. mukaigawae*, 210 from *S. japonicas,* and 60 from *Torymus* sp. The phylogenetic relationship among all *Wolbachia wsp* sequences in *A. mukaigawae* communities were analyzed using neighbor-joining (NJ) algorithm in MEGA and maximum likelihood algorithm (ML) in PAUP. The resulting NJ tree and the ML tree were very similar overall (Figure 2 and Figure S1), and so we only presented the NJ trees here. As shown in the tree, the *A. mukaigawae wsp* sequences formed five distinct clusters, and those of *S. japonicus* formed eight distinct clusters (Figure 2). Each cluster represented a distinct strain, and the result clearly revealed multiple *Wolbachia* infections in both species (Figure 2).

Nine strains based on genetic distance, designated herein as W_1_ to W_9_, were identified in the wasp communities (Table 2 and Figure 2). As described above, the genetic distances between all sequence pairs were calculated in MEGA using Kimura 2-parameter model with Complete Deletion option, and pairs of *Wolbachia* strains with *wsp* genetic distances of 1.5% or greater were considered as distinct strains. The mean pairwise genetic distance of *wsp* sequences is 13.2%, with highest between W_8_ and W_9_ (21.1%) and the lowest between W_4_ and W_6_ (3.0%). Although W_5a_ and W_5b_ did not qualify as distinct strains under the 1.5% genetic distance criterion, W_5b_ differed from W_5a_ by having a distinct 24 bp insertion between positions 418 and 442. In addition, W_6_ was identical to the strain found from *A. mukaigawae* in the two Japanese populations [36].

**Table 2:** Pairwise distances between *wsp* consensus sequences calculated on nucleotide level†.

		W_1_	W_2_	W_3_	W_4_	W_5_		W_6_	W_7_	W_8_	W_9_
						W_5a_	W_5b_				
W_1_											
W_2_		0.082									
W_3_		0.113	0.103								
W_4_		0.050	0.059	0.104							
W_5_	W_5a_	0.190	0.182	0.197	0.188						
	W_5b_	0.182	0.182	0.197	0.188	0.011					
W_6_		0.054	0.054	0.123	0.030	0.180	0.182				
W_7_		0.111	0.066	0.047	0.106	0.207	0.207	0.126			
W_8_		0.158	0.153	0.072	0.164	0.121	0.128	0.175	0.110		
W_9_		0.135	0.098	0.159	0.123	0.196	0.196	0.118	0.154	0.211	

† All gaps were deleted and homogenous patterns among lineages and uniform rates among sites assumed. Kimura 2-parameter model was applied for nucleotides and distance for nucleotides was given below the diagonal.

### Distribution of *Wolbachia* Strains in Different Geographic Populations of *A. mukaigawae* Communities

**Figure 3:**
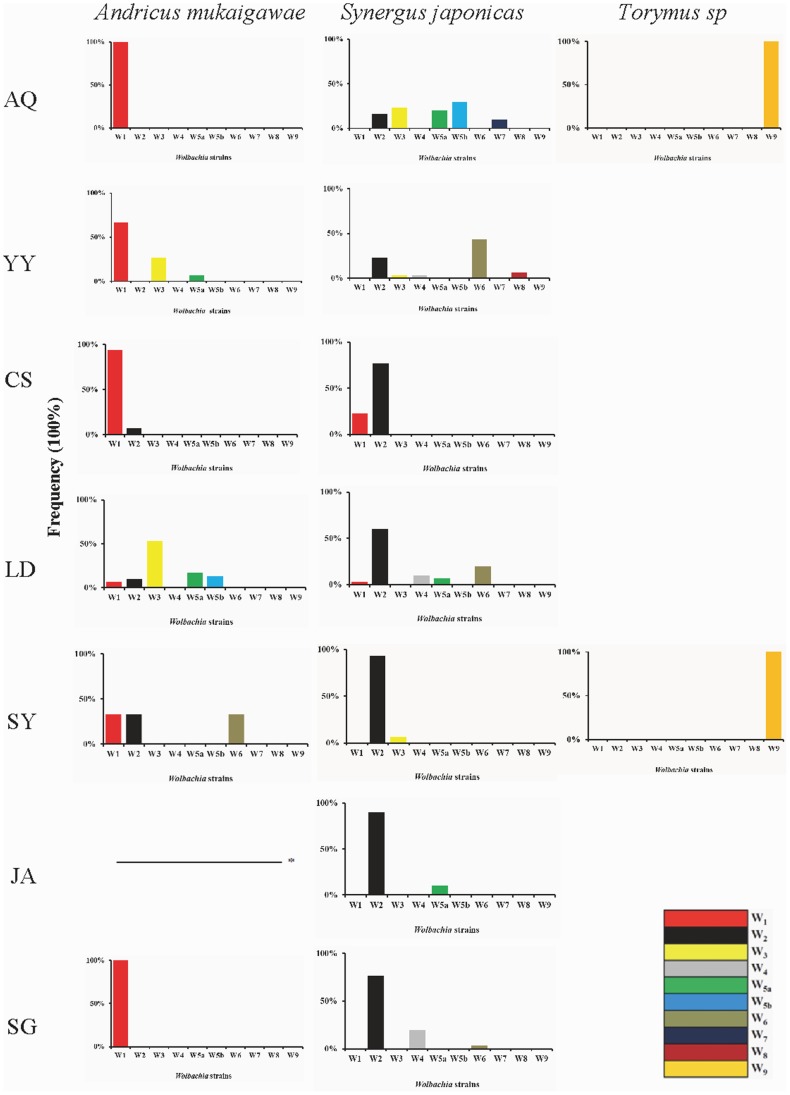
Diversity and distribution pattern of *Wolbachia* strains in three wasp species in different locations. The abbreviations AQ, YY, CS, LD, SY, JA and SG indicate sampled populations shown in Table 1. Dash line (–) indicates no *Wolbachia* infection detected. On the horizontal axis are *Wolbachia strains* and the vertical axis represents the frequency of *Wolbachia* strains in different locations in three wasp species.

The distribution of the nine *Wolbachia* strains in the seven geographic populations of *A. mukaigawae* communities is shown in Figure 3. *A. mukaigawae* was infected with strains W_1_, W_2_, W_3_, W_5a_, W_5b_ and W_6_, of which W_1_ was the most common, infecting all the six populations of *A. mukaigawae* that had *Wolbachia* infection at all. The Anqing and Shaoguang populations had only single infection (W_1_), whereas multiple infections were found in all other *Wolbachia* infected populations - 3 strains (W_1_, W_3_, and W_5a_) in Yueyang population, 2 (W_1_, W_2_) in Changsha, 5 (W_1_, W_2_, W_3_ W_5a_, W_5b_) in Loudi, and 3 (W_1_, W_2_, W_6_) in Shaoyang (Table S1). The inquiline species *S. japonicus* was infected with the *Wolbachia* strains W_1_, W_2_, W_3_, W_4_, W_5a_, W_5b_, W_6_, W_7_ and W_8_, of which W_2_ was the most common, being present in all seven *Wolbachia* infected populations, and W_7_ and W_8_ were the rarest, infecting only the Anqing and Yueyang populations, respectively (Table S2). All *Wolbachia* infected populations of *S. japonicus* had multiple infections - Anqing population was infected with 5 strains (W_2_, W_3_, W_5a_, W_5b_, W_7_), Yueyang with 5 (W_2_, W_3_, W_4_, W_6,_ W_8_), Changsha with 2 (W_1_, W_2_), Loudi with 5 (W_1_, W_2_, W_4_, W_5a_, W_6_), Shaoyang with 2 (W_2_, W_3_), Jian with 2 (W_2_, W_5a_), and Shaoguang with 3 (W_2_, W_4_, W_6_). In contrast, *Wolbachia* in *Torymus* sp. populations were not nearly as diverse - the only detected strain was W_9_, in the Anqing and Shaoyang populations.

Noticeably several *Wolbachia* strains, including W_1_, W_2_, W_3_, W_5a_, W_5b_ and W_6_, were shared by *A. mukaigawae* and *S. japonicas*, being W_1_ and W_2_ in Changsha, W_1_, W_2_ and W_5a_ in Loudi, and W_3_ in Yueyang.

### Recombination of *wsp* Gene between *Wolbachia* Strains in *A. mukaigawae* Communities

Recombination analysis of the aligned *wsp* sequences suggests three recombination events of *Wolbachia* strains in *A. mukaigawae* communities (Table 3, Figure S2–S4). For each recombination event, the breakpoints and probabilities varied depending on the method used, and we only present the result of a method with the highest probability (Figure S2–S4).

**Table 3:** Recombination analysis of *Wolbachia wsp* gene using 6 methods implemented RDP package.

Recombinant	Major parent†	Minor parent	Beginning/Ending breakpoint(in alignment)	Method	P-value
W_8_	W_5a_	W_3_	–	RDP	–
			–	GENECONV	–
			42/339	BootScan	1.260E-10
			42/339	Maxchi	1.100E-13
			–	Chimaera	–
			–	3Seq	
W_6_	W_9_	W_4_	7/254	RDP	3.525E-10
			–	GENECONV	–
			–	BootScan	–
			57/245	Maxchi	4.379E-10
			57/253	Chimaera	2.113E-09
			14/253	3Seq	1.221E-15
W_2_	W_6_	W_7_	–	RDP	–
			120/575	GENECONV	1.060E-08
			120/575	BootScan	7.443E-10
			120/575	MaxChi	9.871E-10
			120/560	Chimaera	4.636E-10
			120/577	3Seq	2.777E-13

† Major parent: parent contributing the larger fraction of sequence; Minor parent: parent contributing the smaller fraction of sequence.

Strain W_8_ was detected as a recombinant by 2 of the 6 used methods: BootScan (P<10^−9^), Machi (P<10^−12^) (Table 3). Its major and minor parents were W_5a_ and W_3_, respectively. BootScan analysis showed it to be more closely related to W_5a_ at the region positions 1–42 (100% identity) and 340–603 (99.6% identity) and more closely related to W_3_ in the recombinant region (positions 43–339) (99.3% identity) (Figure S2).

Strain W_6_ was detected as a recombinant by four methods: RDP (P<10^−9^), Maxchi (P<10^−9^), Chimaera (P<10^−8^), 3Seq (P<10^−14^) (Table 3). The major and minor parents are W_9_ and W_4_, respectively. Chimaera analysis showed it to be more closely related to W_9_ at positions 1–57 (93.0% identity) and 254–564 (100.0% identity) while being more closely related to W_4_ in the recombinant region (positions 58–253) (98.0% identity) (Figure S3).

Strain W_2_ was detected as a recombinant by five methods: GENECONV (P<10^−7^), BootScan (P<10^−9^), Machi (P<10^−9^), Chimaera (10^−9^), 3Seq (P<10^−12^) (Table 3). Its major and minor parents are W_6_ and W_7_, respectively. Results of the GENECONV analysis showed it to be more closely related to W_7_ at the region positions 1–120 (98.3% identity) and 576–585 (100% identity) while being more closely related to W_6_ in the recombinant region (positions 121–575) (99.3% identity) (Figure S4).

## Discussion

The Cynipidae comprises about 1400 described species and is the second most species-rich group of gall inducers after the gall midge family Cecidomyiidae (Diptera) [39], [51]. Recent studies have revealed *Wolbachia* infection in diverse cynipid species, including 4 species in Aylacini, 8 in Cynipini, 11 in Diplolepidini and 6 in Synergini [9], [33]–[38]. From 8 locations in southern China where we collected the unisexual galls, high levels of *Wolbachia* infection were revealed in the insect communities associated with these galls, occurring in 6 out of 8, 7 out of 7, and 2 out of 2 populations of *A. mukaigawae*, *S. japonicas*, and *Torymus* sp, respectively.

### Multiple Infections of *Wolbachia* in *A. mukaigawae* and *S. japonicus*

The high levels of multiple *Wolbachia* infections found in *A. mukaigawae* and its associated inquiline *S. japonicus*, with 5 and 8 *Wolbachia* strains, respectively, are rather noticeable and unheard of in cynipid wasps [34]. In fact, the high levels of multiple infections comparable to what we found in the *A. mukaigawae* associated communities are infrequent among insects in general, although it is relatively common for host insects to be infected with two or three *Wolbachia* strains [5], [52]. The only other species known to us with similar high levels of multiple infections of *Wolbachia* include 3 species of ants, *i.e., Doronomyrmex pacis* (infected with 6 strains) [53], *Acromyrmex octospinosus* (4 strains) [8] and *Formica exsecta* (5 strains) [7], and one species of tephritid fruitfly *Bactrocera ascita* (5 strains) [54]. This phenomenon may be attributable to the increased possibility of horizontal transmission and gene recombination due to the special niche relationship between the gall inducer and the inquiline.

### Frequent Recombination Events of *Wolbachia* in the *A. mukaigawae* Communities

The three recombinant strains identified in our study and their respective presumed parent strains (in parenthesis) are W_8_ (W_3_ & W_5a_), W_2_ (W_6_ & W_7_)_,_ and W_6_ (W_4_ & W_9_) (Figure S2–S4) (Table 3). For each of W_8_ (W_3_ & W_5a_) and W_2_ (W_6_ & W_7_) families, the recombinant strain and both parent strains were all found in some populations of *S. japonicus*, while no single population of *S. japonicus* were found to harbor all three of either of the two recombinant families. This may be due to potential historical losses and relatively low frequency of the recombinants and/or the parent strains in natural populations. For W_6_ (W_4_ & W_9_) recombinant family, only the recombinant strain W_6_ and the parent strain W_4_ were detected in *S. japonicus*; the other parental strain W_9_ was only detected in the parasitic wasps *Torymus* sp. Several studies have showed it is possible for *Wolbachia* to be horizontally transferred between hosts and their associated parasitoids [5], [13], [55]. Since parasitoid attack is normally fatal [56], the direction of *Wolbachia* horizontal transmission should be unidirectional from host to parasitoid. In our system, W_9_ may have historically infected *S. japonicus*, and subsequently been transferred from *S. japonicus* to *Torymus* sp. after a recombination event with W_4_. We did not detect W_9_ in *S. japonicus*, either because of potentially historical loss. Overall, our study for the first time provides molecular evidence supporting *wsp* gene recombination of *Wolbachia* in cynipid gall wasps, and is in line with findings of recent studies on *Wolbachia* infecting other arthropod groups as diverse as ants [7], Fig wasps [57] and spiders [58].

Recombination plays an important role in the evolution of bacteria in general and *Wolbachia* in particular, in ways similar to sexual reproduction of the majority of higher animals and plants [59]. Vertical transmission, the main transmission mode of *Wolbachia*, results in the accumulation of deleterious mutations while providing a stable and effective way of transmission. Recombination, on the other hand, can create new recombinants, resulting in strains without the deleterious mutations and increased genetic diversity of *Wolbachia* strains, allowing them to utilize diverse hosts, a phenomenon well documented in pathogenic bacteria [60], [61].

### Plant-mediated Horizontal Transmission of *Wolbachia* between *A. mukaigawae* and *S. japonicus*

Some host species with multiple *Wolbachia* strains may acquire additional infections through horizontal transmission [13], [54]. In the wasp communities associated with the unisexual galls of *A. mukaigawae*, two mechanisms are probably involved with the horizontal transmission of *Wolbachia*: host-parasitoid interactions and contact with same plant tissues. Evidences for horizontal transmission of *Wolbachia* between *A. mukaigawae* and *S. japonicus* come from the fact that several *Wolbachia* strains, *i.e.,* W_1_, W_2_, W_3_, W_5a_, W_5b_ and W_6,_ were found in both species, and in some communities, multiple identical strains were detected in both *A. mukaigawae* and *S. japonicus*, *e.g.,* W_1_ and W_2_ in Changsha, W_1_, W_2_ and W_5a_ in Loudi, and W_3_ in Yueyang (Figure 2 and Figure 3). Our results are consistent with that of a previous study, showing possible horizontal transmission of *Wolbachia* between gall-inducers and their associated inquilines [9].

Plant-mediated horizontal transmission of *Wolbachia* between host insect species was reported by several studies [23], [24]. Caspi-Fluger *et al* [25] found that insect symbiotic *Rickettsia* were transferred from whiteflies to the phloem of the host plants, and could be acquired by other whiteflies. Both the gall-inducer and its associated inquiline are phytophagous in the larval stages of their development, feeding on galls, which are modified live plant structure [31]. In our system, larvae of *A. mukaigawae* feed on the nutritive tissue lining the interior of its larval chamber in the center of the gall while larvae of the inquilinous *S. japonicus* feed in their own larval chambers made in gall tissue surrounding the central larval chamber of the *A. mukaigawae* [43]. During gall induction and development, plant tissues regularly come in contact with the insect secretions including surface secretions from eggs or young larvae, ovipositional fluid, frass, and/or saliva [62]. At the same time, *Wolbachia* are distributed in insect genital tissues, somatic tissues [63], [64], salivary glands [65], and hemolymph [66]. Therefore, it is highly likely that *Wolbachia* are transferred between gall wasps and inquiline wasps by way of gall tissues.

We are aware that the larvae of the *S. japonicus* grow faster than that of the *A. mukaigawae* and eventually the larvae of *A. mukaigawae* cease to develop [67]. Consistent with the previously studies [67], we did not find a single gall where larvae chambers of *A. mukaigawae* and *S. japonicus* coexisted in our dissection of galls (Table 1). In this case, the direction of plant-mediated horizontal transmission should be unidirectional from *A. mukaigawae* to *S. japonicus*.

Inquiline gall wasps do not always kill their hosts and an extensive parenchyma peripheral to the larval chamber in some host galls may provide extra space for the inquiline cells [31]. We suggested that if some host galls provide enough space for larvae of both *S. japonicus* and *A. mukaigawae*, inquilines (*S. japonicus*) and gall inducers (*A. mukaigawae*) may emerge from a single gall. When this happens, plant-mediated horizontal transmission between the inquiline and the gall inducer should be limited bidirectional.

This may explain the high levels multiple *Wolbachia* infections of *A. mukaigawae* and *S. japonicus*, especially the fact that *A. mukaigawae* was found to be infected with as many as 5 strains of *Wolbachia*.

### Horizontal Transmission of *Wolbachia* by Host-parasitoid Association

Although we do not have direct evidence to show whether *Torymus* sp. parasitizes *A. mukaigawae or S. japonicus*, it seems more likely that the parasitoid species attacks the inquiline species because the two share a same *Wolbachia* strain, W_9_. As discussed above, the strain may have historically infected *S. japonicus* and subsequently been transferred to *Torymus* sp. through host-parasitoid interactions. Since parasitoid attack is normally fatal, horizontal transmission between *S. japonicus* (host) and *Torymus* sp. (parasitoid) is likely to be unidirectional. Nonetheless, we only had limited success in rearing the parasitoid species and further work in the future is certainly needed to elucidate a possibly much complex relationship among *Torymus* sp. and its host species *S. japonicus,* and possibly *A. mukaigawae* as well, and the horizontal transmission of *Wolbachia* between them.

## Conclusions

In conclusion, the present study found high levels of multiple *Wolbachia* infections in *A. mukaigawae* and *S. japonicus* associated with the unisexual galls of *A. mukaigawae*. Our results suggested the potentialities that the horizontal transmission of *Wolbachia* was plant-mediated, and revealed multiple recombination events for *Wolbachia* in gall associated wasp communities. We further suggest that recombination events and potential plant-mediated horizontal transmission play an important role in the high levels of multiple *Wolbachia* infection of *A. mukaigawae* and *S. japonicus.*

## Supporting Information

Figure S1 **Maximum Likelihood tree for** ***Wolbachia*** **strains of** ***A. mukaigawae*** **,** ***S. japonicus*** **and** ***Torymus*** **sp. based on** ***wsp*** **sequence.** W_1_–W_9_ indicate *Wolbachia* strains in *A. mukaigawae* communities. The abbreviations AQ, YY, CS, LD, SY, JA and SG in parentheses indicate the populations shown in Table 1. The number following AQ, YY, CS, LD, SY, JA and SG indicate the amount of *Wolbachia* strains per population. Numbers above branches were bootstrap values computed from 1000 replications. *Wolbachia* from *Trichogramma semblidis* was used as outgroup.
(TIFF)

Figure S2 **Recombination events of** ***wsp*** **gene between** ***Wolbachia*** **strains W_5a_ and W_3_ by BootScan method.** Percentages around the sequence alignments show the similarities of the daughter sequence to its major or minor parent sequences (marked with the same background color). Major parent: parent contributing the larger fraction of sequence; minor parent: parent contributing the smaller fraction of sequence.
(TIF)

Figure S3 **Recombination events of** ***wsp*** **gene between** ***Wolbachia*** **strains W_9_ and W_4_ by Chimaera method.**
(TIF)

Figure S4 **Recombination events of** ***wsp*** **gene between** ***Wolbachia*** **strains W_6_ and W_7_ by GENECONV method.**
(TIF)

Table S1 The distribution of *Wolbachia* strains in each individual from different geographic populations of *A. mukaigawae.* ^†^The abbreviations AQ, YY, CS, LD, SY and SG indicate the populations shown in Table 1. The number following AQ, YY, CS, LD, SY and SG indicate different individuals from the same population. ^‡^W_1_–W_6_ indicate *Wolbachia* strains in *A. mukaigawae*. ^§^The number indicate the amount of *Wolbachia* strains in each individual of *A. mukaigawae.*
(DOC)

Table S2 The distribution of *Wolbachia* strains in each individual in geographic population of *S. japonicus.* ^†^The abbreviations AQ, YY, CS, LD, SY, JA and SG indicate the populations shown in Table 1. The number following AQ, YY, CS, LD, SY, JA and SG indicate different individuals from the same population. ^‡^W_1_–W_8_ indicate *Wolbachia* strains in *S. japonicus*. ^§^The number indicate the amount of *Wolbachia* strains in each individual of *S. japonicus.*
(DOC)
