# Supplementary figures and images for: High Levels of Multiple Infections, Recombination and Horizontal Transmission of Wolbachia in the Andricus mukaigawae (Hymenoptera; Cynipidae) Communities
Source: PLoS One. 2013 Nov 8;8(11):e78970. doi: 10.1371/journal.pone.0078970 (PMC3826730; doi:10.1371/journal.pone.0078970)

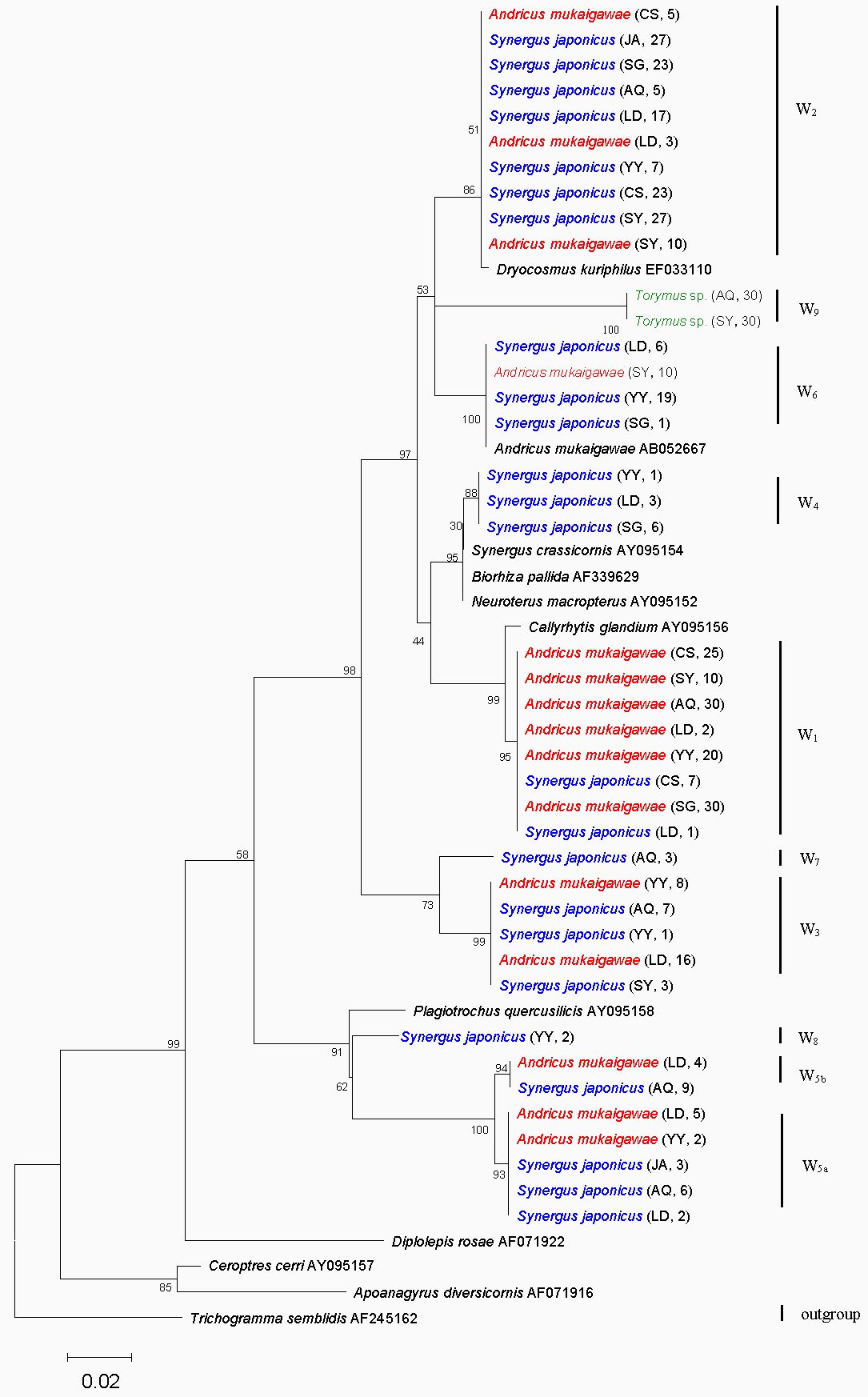

Supplement: Figure S1 — Maximum Likelihood tree for Wolbachia strains of A. mukaigawae , S. japonicus and Torymus sp. based on wsp sequence. W1–W9 indicate Wolbachia strains in A. mukaigawae communities. The abbreviations AQ, YY, CS, LD, SY, JA and SG in parentheses indicate the populations shown in Table 1. The number following AQ, YY, CS, LD, SY, JA and SG indicate the amount of Wolbachia strains per population. Numbers above branches were bootstrap values computed from 1000 replications. Wolbachia from Trichogramma semblidis was used as outgroup. (TIFF) [file pone.0078970.s001.tiff]

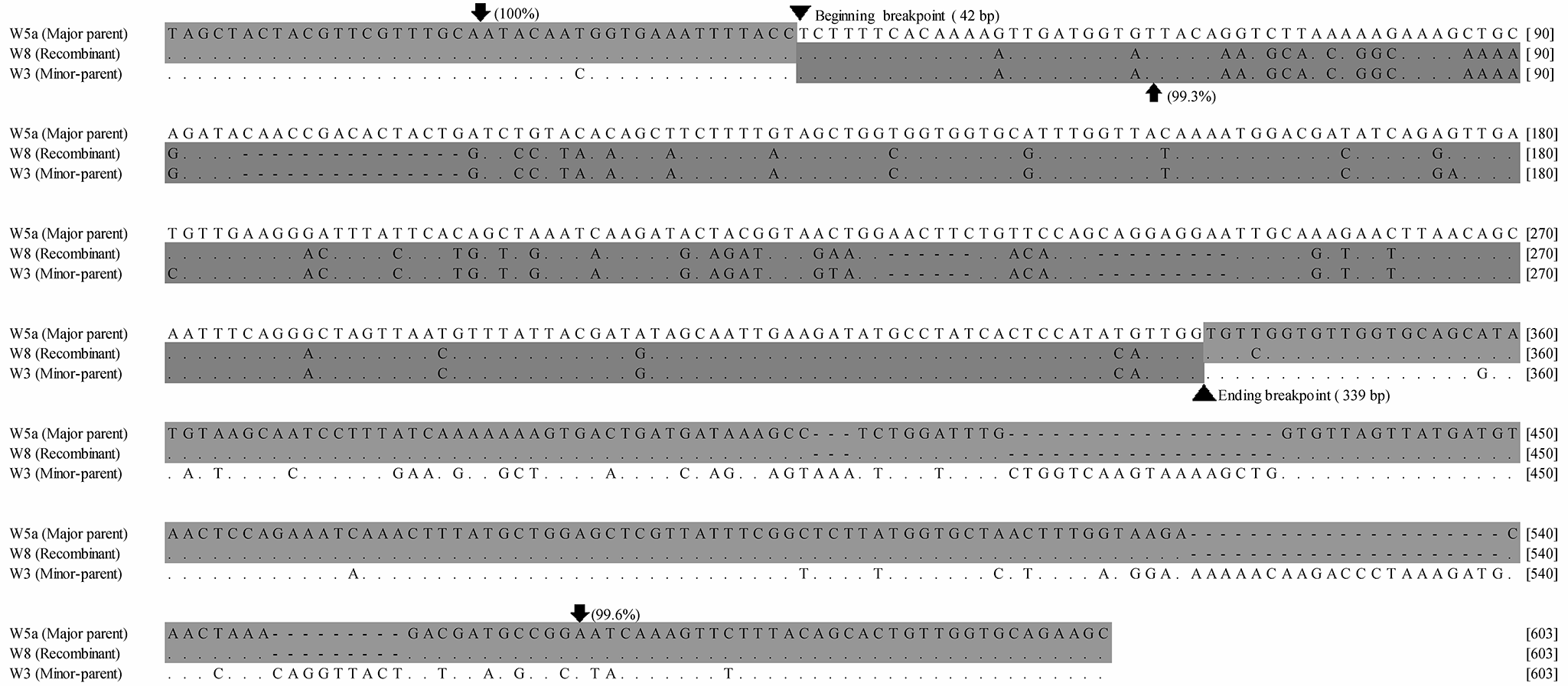

Supplement: Figure S2 — Recombination events of wsp gene between Wolbachia strains W5a and W3 by BootScan method. Percentages around the sequence alignments show the similarities of the daughter sequence to its major or minor parent sequences (marked with the same background color). Major parent: parent contributing the larger fraction of sequence; minor parent: parent contributing the smaller fraction of sequence. (TIF) [file pone.0078970.s002.tif]

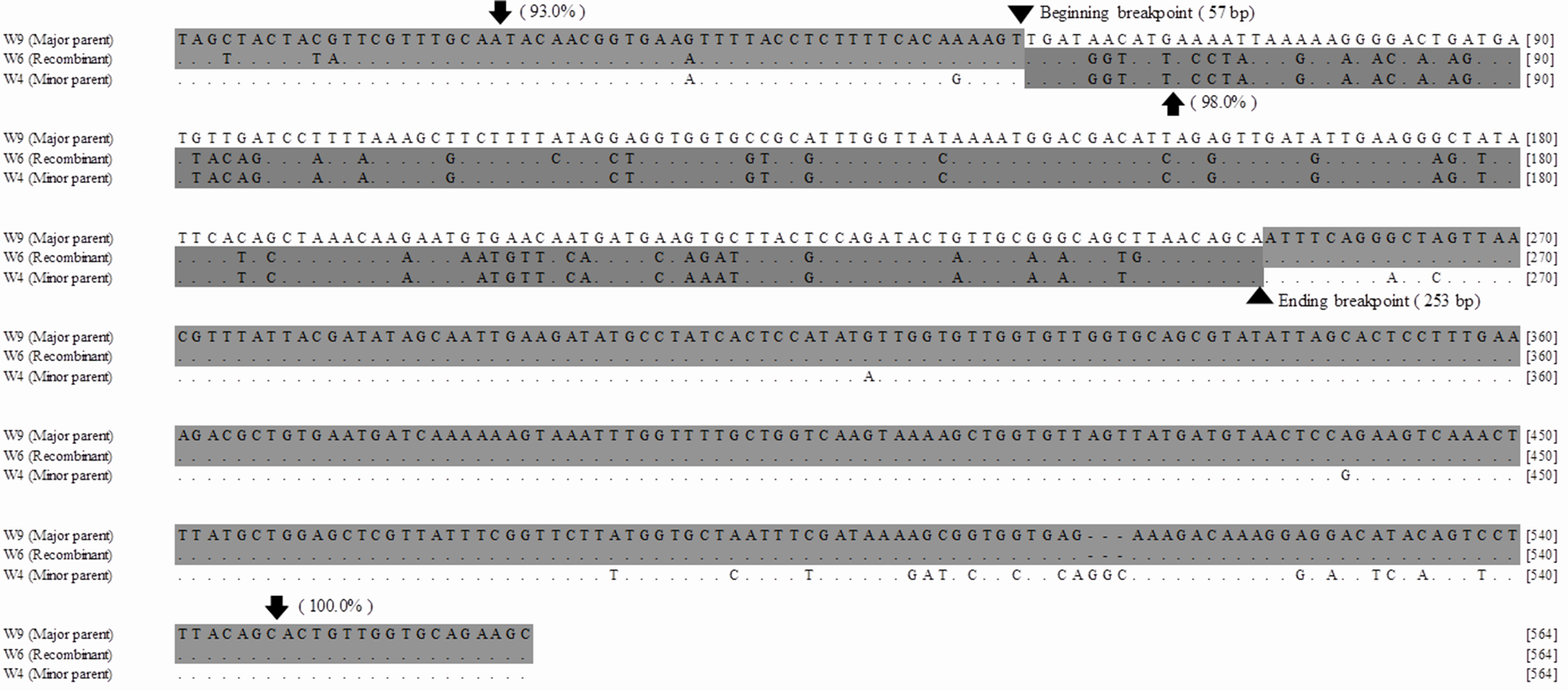

Supplement: Figure S3 — Recombination events of wsp gene between Wolbachia strains W9 and W4 by Chimaera method. (TIF) [file pone.0078970.s003.tif]

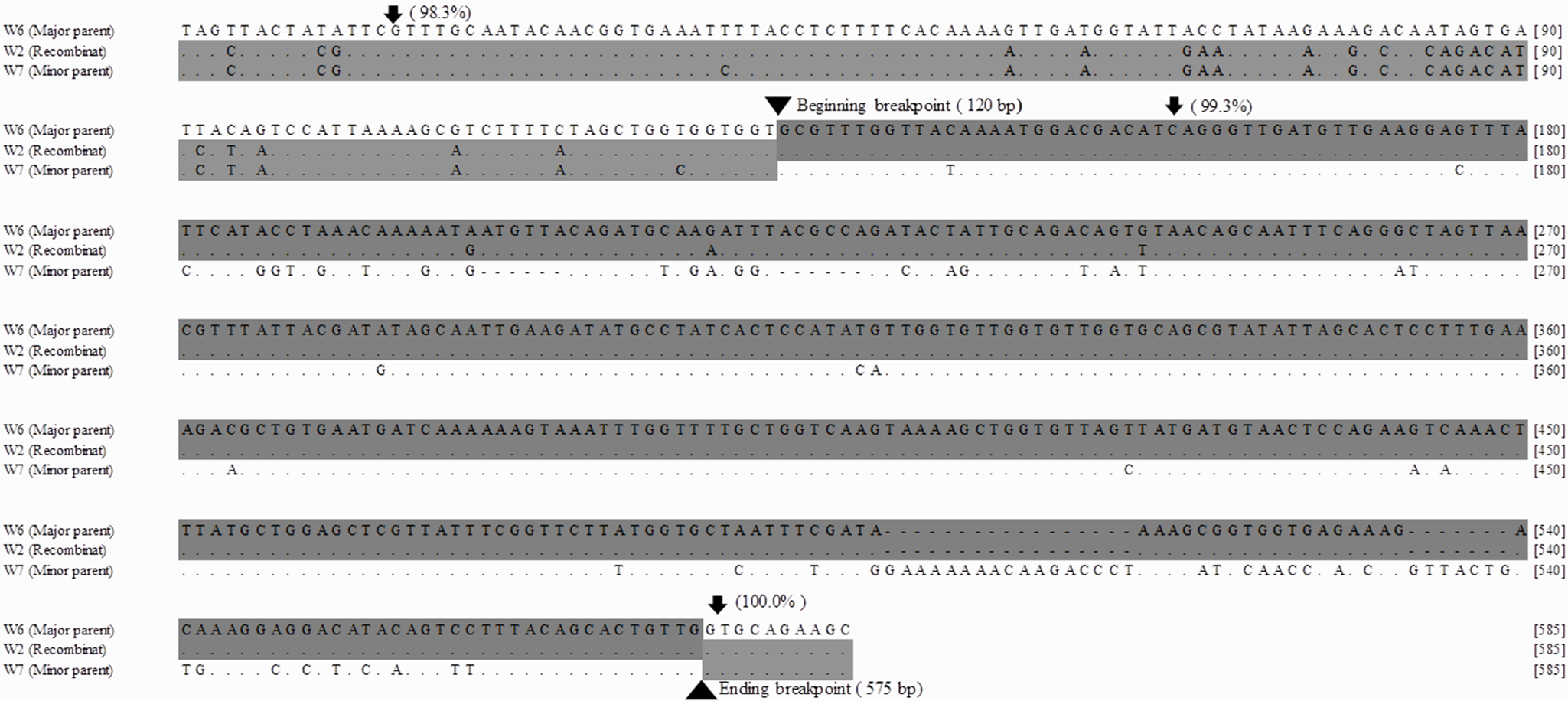

Supplement: Figure S4 — Recombination events of wsp gene between Wolbachia strains W6 and W7 by GENECONV method. (TIF) [file pone.0078970.s004.tif]
