# Supplementary material for: High Levels of Multiple Infections, Recombination and Horizontal Transmission of Wolbachia in the Andricus mukaigawae (Hymenoptera; Cynipidae) Communities
Source: PLoS One. 2013 Nov 8;8(11):e78970. doi: 10.1371/journal.pone.0078970 (PMC3826730; doi:10.1371/journal.pone.0078970)
Supplement: Table S2 — The distribution of Wolbachia strains in each individual in geographic population of S. japonicus. †The abbreviations AQ, YY, CS, LD, SY, JA and SG indicate the populations shown in Table 1. The number following AQ, YY, CS, LD, SY, JA and SG indicate different individuals from the same population. ‡W1–W8 indicate Wolbachia strains in S. japonicus. §The number indicate the amount of Wolbachia strains in each individual of S. japonicus. (DOC) [file pone.0078970.s006.doc]

|  | AQ^†^-1 | AQ-2 | AQ-3 | YY-1 | YY-2 | YY-3 | CS-1 | CS-2 | CS-3 | LD-1 | LD-2 | LD-3 | SY-1 | SY-2 | SY-3 | JA-1 | JA-2 | JA-3 | SG-1 | SG-2 | SG-3 |
| --- | --- | --- | --- | --- | --- | --- | --- | --- | --- | --- | --- | --- | --- | --- | --- | --- | --- | --- | --- | --- | --- |
| W_1_^‡^ |  |  |  |  |  |  | 1 | 3 | 3 |  |  | 1 |  |  |  |  |  |  |  |  |  |
| W_2_ |  |  | 5 |  | 7 |  | 9 | 7 | 7 | 7 | 5 | 6 | 10 | 8 | 10 | 7 | 10 | 10 | 8 | 9 | 6 |
| W_3_ | 2^§^ | 3 | 2 | 1 |  |  |  |  |  |  |  |  |  | 2 |  |  |  |  |  |  |  |
| W_4_ |  |  |  |  | 1 |  |  |  |  |  | 3 |  |  |  |  |  |  |  | 2 |  | 4 |
| W_5a_ | 6 |  |  |  |  |  |  |  |  |  | 2 |  |  |  |  | 3 |  |  |  |  |  |
| W_5b_ | 2 | 7 |  |  |  |  |  |  |  |  |  |  |  |  |  |  |  |  |  |  |  |
| W_6_ |  |  |  | 9 |  | 10 |  |  |  | 3 |  | 3 |  |  |  |  |  |  |  | 1 |  |
| W_7_ |  |  | 3 |  |  |  |  |  |  |  |  |  |  |  |  |  |  |  |  |  |  |
| W_8_ |  |  |  |  | 2 |  |  |  |  |  |  |  |  |  |  |  |  |  |  |  |  |
